# Existing and emerging pharmacological approaches to the treatment of mania: A critical overview

**DOI:** 10.1038/s41398-022-01928-8

**Published:** 2022-04-23

**Authors:** Giulio Sparacino, Norma Verdolini, Eduard Vieta, Isabella Pacchiarotti

**Affiliations:** 1 Bipolar and Depressive Disorders Unit, Institute of Neuroscience, Hospital Clinic, University of Barcelona, IDIBAPS, Barcelona, Spain; 2 Department of Health Sciences, Università degli Studi di Milano, Milan, Italy; 3 Biomedical Research Networking Center on Mental Health (CIBERSAM), Barcelona, Spain

**Keywords:** Bipolar disorder, Schizophrenia

## Abstract

Manic episodes are a defining, frequent and dramatically disabling occurrence in the course of Bipolar Disorder type I. Current pharmacotherapy of mania lists a good number of agents, but differences in efficacy and safety profiles among these agents must be considered in order to tailor personalized therapies, especially when the long-term course of the illness is considered. There is wide room and need to ameliorate current pharmacological approaches to mania, but ongoing pharmacological research on the topic is scant. In this work we try to critically assess clinical factors and patients’ characteristics that may influence the treatment choice for manic episodes. In addition, we conduct a narrative review on experimental pharmacology of bipolar mania and psychotic disorders, presenting a critical overview on agents which could represent treatment alternatives for a manic episode in the next future. Results show limited novel or ongoing research on agents acting as mood stabilizers (Ebselen, Valnoctamide and Eslicarbazepine did not reach statistical significance in demonstrating antimanic efficacy). As for the emerging experimental antipsychotic, some of them (including KarXT, SEP-363856, RO6889450, ALKS3831) have demonstrated good antipsychotic efficacy and a favorable safety profile, but little is known about their use in patients with bipolar disorder and specifically designed trials are needed. Lastly, some benefits for the treatment of mania could be expected to come in the next future from non-mood stabilizers/non-antipsychotic agents (especially PKC inhibitors like Endoxifen): long-term trials are needed to confirm positive results in terms of long-term efficacy and safety.

## Introduction

Bipolar Disorders (BDs) are a group of severe mood disorders with a chronic and recurrent course, characterized by periods of euthymia alternated with manic/hypomanic, depressive with or without the presence of mixed features and mixed episodes [1]. BDs rank as the 17th leading source of disability among all diseases worldwide with the highest suicide rate among all psychiatric conditions, being 20–30 times higher than in the general population [1]. Within BDs, manic episodes (MEs) affect overall more than 1% of the general population [2]. The presence of a manic episode is the required criterion needed for the diagnosis of BD type I (BD-I).

The 5th and last edition of the Diagnostic and Statistical Manual of Mental Disorders (DSM-5) has made changes to the “criterion A” for a ME, which now requires a distinct period of abnormally and persistently elevated, expansive, or irritable mood and abnormally and persistently increased activity or energy present most of the day, almost every day for minimum 1 week (or less if patient requires hospitalization) [3]. According to the DSM-5 definition, a ME must be accompanied by marked impairment in psychosocial functioning, require hospitalization, and/or involve psychotic symptoms. Another difference between DSM-5 and its prior editions is that a diagnosis of BD-I can now be made in patients in whom the ME emerges while being treated (i.e., with medication or ECT) for major depression, when a fully syndromic ME persists beyond the expectable effect of the treatment. Moreover, in DSM-5 the diagnosis of “mixed episode” no longer exists, and has been replaced with the more dimensional “with mixed features” specifier that can be applied to mania or hypomania. The DSM-5 included other specifiers that can accompany a manic episode, such as anxious distress, rapid cycling, mood-congruent or mood-incongruent psychotic features, catatonia, peripartum onset, and seasonal pattern. As for latest version of the International Classification of Diseases (ICD-11), which from 2022 should be in worldwide use, the definition of a ME is now almost identical with the DSM-5 as regards entry criteria, duration, hospitalization, and the presence or absence of psychotic features and impairment in social and occupational functioning, although with some formal and methodological dissimilarities (e.g. ICD-11’s descriptions of the “essential features” of mood episodes are not presented as equivalents of strict diagnostic criteria and symptom counts or duration cut-offs are generally absent) [4–6].

From a clinical point of view, one first interesting point to be remarked is that a ME actually represents an heterogenous entity. In fact, the DSM-5 recognizes up to 7 clinical specifiers for a ME and each of these could require a different therapeutic approach in terms of prevention, early intervention, pharmacotherapy, psychotherapy, psychoeducation and rehabilitation.

Another important point to consider is that a ME needs to be understood and treated not in a “cross-sectional” way, but as a phenomenon included in the complex and long-lasting course of bipolar illness. As a consequence, the treatment of the acute manic phase should already consider the maintenance treatment and course specifiers, such as the number of episodes, predominant polarity, the presence of rapid cycling, the risk of counterpolar switch [7], making the pharmacological treatment a complex choice. Predominant polarity (PP) is a course specifier concept aimed at identifying the presence of a predominant affective pole of illness within the same patient across its illness course [8]. Data on manic PP in the course of BD-I is conflicting since it ranges from 11.5 to 45.7% across studies [9, 10] mainly due to variation of temporal criteria used for defining PP. Strictly connected with PP is the concept of Polarity Index (PI), a metric used to orient pharmacotherapy in BD on the basis of PP which will be discussed in the next section.

Other clinical aspects that should be considered when treating a ME include the management of psychomotor agitation; the differences in the manic symptoms presentation in order to personalize treatment for a specific patient as much as possible (precision psychiatry); the need for using monotherapy versus combination therapy; the presence of mood incongruent psychotic symptoms which will require a more complex treatment; the importance of addressing compliance issues and the possibility of choosing long-acting formulations. Lastly, safety/tolerability issues in the short and long-term treatment, the presence of psychiatric/medical comorbidities and the need for a functional recovery complete this complex scenario [7, 11].

As for the biological bases, multiple factors interact in the complex equation of the pathophysiology of a ME, such as neuro-hormonal pathways, neurotransmission, signal transduction, regulation of gene expression, oxidative stress, neuroplasticity, changes in the immune system, psychosocial, developmental and environmental stress events and the severity of the episode [12]. Overall, these factors could represent specific pharmacological targets.

The aim of this paper is to critically assess clinical factors and patients’ characteristics that may influence the treatment choice in mania, as well as review the current, evidence-based pharmacological treatments for mania. Finally, we will discuss emerging and experimental pharmacological options which could represent useful alternative in treating a manic episode in the near future.

## Method

We conducted a narrative, non-systematic review of literature about (i) current pharmacotherapy of bipolar mania, (ii) novel and experimental pharmacotherapy of bipolar mania, and (iii) novel and experimental pharmacotherapy of psychotic disorders. For this purpose, in May 2021 we conducted a series of targeted literature searches in PubMed, clinicaltrials.gov and clinicaltrialsregister.eu. Key words for the search—pharmacological treatment/drugs therapy/pharmacotherapy of bipolar mania, psychotic disorders and schizophrenia—were identified by our team of experts. The need for including key words related to psychotic disorders was motivated by both: (a) many current pharmacological approaches to mania sharing indication for psychotic disorders and (b) the paucity of ongoing experimental pharmacology studies specifically dedicated to mania and the consequent need for looking at the richer field of psychotic disorders experimental pharmacology, wherefrom many drugs have come in the last decades which are currently used for treating MEs.

Only articles written in English language and published in the last ten years (May 2011–May 2021) were searched. All abstracts were checked by the authors and all kinds of study design were critically taken into consideration. Also, some papers identified in the reference list of the papers from the original search were considered. The results were then synthesized and incorporated with clinical experience of the authors.

## Results

### Existing approaches to the treatment of mania

The existing approaches for the treatment of MEs are summarized and categorized in Table 1. A tentative treatment algorithm can be found in a recently published work from our same research group [7].

**Table 1:**
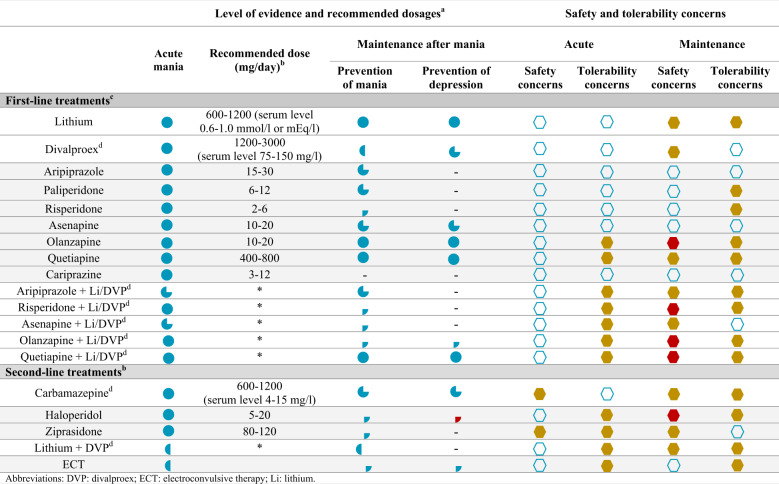
Current first-line and second-line options for the treatment of acute mania.

*DVP* divalproex, *ECT* electroconvulsive therapy, *Li* lithium.

^a^Strength of evidence base for the efficacy of agents used to treat mania:  level 1 evidence: meta-analysis with narrow confidence interval or replicated double-blind (DB), randomized controlled trial (RCT) placebo or active-controlled (*n* ≥ 30 in each active treatment arm);  level 2 evidence: meta-analysis with wide confidence interval or one DB RCT, placebo or active-controlled (*n* ≥ 30 in each active treatment arm);  level 3 evidence: at least one DB RCT placebo or active-controlled (*n* = 10–29 in each active treatment arm) or health system administrative data;  level 4 evidence: uncontrolled trial, anecdotal reports or expert opinion;  level 1 negative evidence;  level 2 negative evidence;  level 3 negative evidence;  level 4 negative evidence; -: no data;  limited or minor impact on treatment selection;  moderate impact on treatment selection;  significant impact on treatment selection.

^b^Doses are reported as per studies.

^c^Treatments are listed by drug class: mood stabilizers, antipsychotics and combination treatments. In each subsection, the recommendations follow a hierarchical order (i.e. lithium before divalproex, aripiprazole before paliperidone, etc). Although monotherapies are listed above combination therapies in the hierarchy, polytherapy may be indicated as the best choice in patients with severe manic episodes and/or a previous history of partial response to monotherapy.

^d^Divalproex and carbamazepine should be avoided in women of childbearing age.

*same recommended doses as monotherapy for each individual treatment.

### Lithium

Lithium still represents the cornerstone of BD treatment. Actually, it ranks as a first-line option for Mania in most current clinical guidelines, both as monotherapy and in combination [7, 11, 13]. Moreover, together with quetiapine, lithium is the only first-line agent which also presents a level 1 evidence of efficacy for prevention of both manic and depressive episodes in the maintenance treatment [13]. Moreover, lithium is usually considered to have a better tolerability profile than quetiapine, especially in the short-term, due to its lack of sedative, hypotensive and direct neurological side-effects [11, 14]. Widely recognized pros of lithium therapy are also its intrinsic antisuicidal and neuro-protective properties [7, 11]. Conversely, the often-cited cons of lithium therapy include a relatively narrow therapeutic index, and the long-term side effects especially on kidney and thyroid function, thus requiring periodic monitoring and blood testing. In terms of a personalized approach, several authors agree in identifying those patients with clinical features that are indicative of a good response to lithium treatment, such as a more classic grandiose or euphoric mania, with fewer prior episodes, with a mania-depression-euthymia course, and those with a family history of BD [7, 11].

### Valproic acid (VPA) and carbamazepine (CBZ)

Both these anticonvulsants have been approved by regulatory agencies for the treatment of MEs, having a level 1 evidence of efficacy for Mania according to the latest guidelines of the Canadian Network for Mood and Anxiety Treatments and International Society for Bipolar Disorders (CANMAT/ISBD) [11]. Nonetheless, in terms of recommendation grade, only VPA is listed as a first-line agent, mainly because of the safety and tolerability issues associated with the treatment with CBZ. Regarding the clinical indications in mania, VPA and to a less extent CBZ, should be prescribed when lithium is not recommended, such as in previous lithium non-response, renal failure, severe dermatological disease, severe gastrointestinal intolerance, cardiovascular insufficiency, Addison’s disease or/and untreated hypothyroidism. Moreover, they could be indicated in BD patients with predominant irritable or dysphoric mood, comorbid substance abuse, multiple prior episodes of illness and/or a history of head trauma [7, 15–19] Of note, due to serious teratogenic effects, both VPA and CBZ are contraindicated in women of childbearing age [20, 21].

### Second generation antipsychotics (SGAs)

The majority of SGAs, such as quetiapine, asenapine, olanzapine, ziprasidone, aripiprazole, risperidone, and cariprazine are also approved by regulatory agencies in United States and Europe as monotherapy for the treatment of mania. With the exception of ziprasidone, all the above-mentioned SGAs are recommended as first-line option for MEs with a level 1 evidence and comparable efficacy to lithium and anticonvulsants. Only the role of olanzapine as a first-line treatment option is controversial, mainly because of long-term safety and tolerability metabolic issues [13]. Despite this, clinical literature and guidelines often agree on the point that SGAs’ indication as monotherapy in MEs only -and partially- applies if the diagnostic possibility of schizoaffective disorder with manic symptoms is considered [7, 11]. On the other hand, the use of SGAs in combination with lithium or divalproex can be actually considered as first-line in MEs with higher severity index, whenever a response is needed faster, in patients with mood-incongruent psychotic or mixed features and/or in those with a previous history of partial response to monotherapy (this is valid except for paliperidone and ziprasidone, whose trials as adjunctive therapies to lithium/divalproex showed lack of efficacy, maybe because of trials’ methodological problems) [11]. Additionally, combination treatment should be considered if no sufficient clinical response is observed after 1–2 weeks with therapeutic doses of a first-line antimanic agent, after dose-optimizing has been performed, non-adherence issues identified and addressed and consideration given to possible substance abuse [11]. Second and third-line choices should be taken in consideration only after multiple trials with adequate dosage of first-line options.

When poor adherence is identified as one of the main reasons for partial or no-response, a long-acting (LA) formulation of SGAs should be offered and, whenever possible, discussed with patients and/or their caregivers as a treatment option. Among SGAs, only Aripiprazole LA formulation is currently labelled as a treatment option for maintenance therapy in BD both in the United States and in Europe, since it has been found effective in delaying manic recurrences without inducing depressive episodes [22]. Other LA SGAs have demonstrated efficacy in treating and preventing manic episodes, with a better level of evidence for risperidone LA, which is in fact approved for maintenance therapy in BD-I in the United States but not in Europe. Nonetheless, neither risperidone LA nor paliperidone LA proved effective in the treatment and prevention of depressive episodes or depressive recurrences in BD, so their use as first-line treatment should be limited to BD patients with manic predominant polarity [23–26]. More broadly, the polarity index of a drug—a RCT-based metric to guide maintenance treatment choice in BD [27]—can be useful to appraise clinical differences among SGAs and tailor personalized therapies [7, 27].

Regarding the use of SGAs as maintenance treatment, it is important to remark that not all SGAs share the same level of evidence for efficacy as maintenance treatment, with only quetiapine classified as level 1 and many in need of further studies to assess efficacy in the long-term [13] (see Table 1). Lastly, it seems important to remark that SGAs are generally considered to represent an advance compared to First Generation Antipsychotics (FGAs), not much on the efficacy side since they share similar level of evidence on this point, but rather for their safety/tolerability profile, avoiding the severe neurological side effects of FGAs, both in the short and the long term. Nevertheless, the use of SGAs is still loaded with important side effects, which somehow shifted on the metabolic side, i.e. weight gain and metabolic syndrome above all, especially for agents like olanzapine. Moreover, some of the old tolerability problems of FGAs, i.e. hyperprolactinemia for risperidone and paliperidone or sedation for quetiapine, whether attenuated are also displayed by SGAs [28]. Thus, it is important to keep in mind that there is extensive room for improvement in the field of pharmacology of BD, especially when considering long-term use after treating an acute ME.

Among FGAs, haloperidol presents a long-known efficacy in the treatment of acute mania and it is still classified as level 1 evidence (see Table 1), but the risk for counterpolar depressive switch and its poor safety/tolerability profile suggests its use as a second-line option or use in special populations, such as manic pregnant women due to its long-known reproductive safety [7].

In the case of a refractory ME, a pharmacogenetic study of cytochrome P450 enzymes (especially 2D6 and 3A4) could be indicated to exclude the eventuality of rapid/ultra-rapid metabolizers. Finally, Clozapine [29] and Electro-Convulsive Therapy (ECT) [30–32] are the main treatment options to be considered in refractory ME, when all the above-mentioned options are not effective.

Indications for current treatment options in special populations (children and adolescents, older-age, childbearing and breastfeeding women) can be found in all major guidelines and recent works on this topic [7, 11, 13].

## Emerging approaches to the treatment of mania

We divided this chapter in four sub-sections: (i) experimental mood-stabilizers (MSs), (ii) re-labelling antipsychotics (APs), (iii) experimental APs and (iv) non-MS/non-AP agents. Emerging approaches for the treatment of Mania are summarized and categorized in Table 2.

**Table 2:** Novel and experimental agents.

Agent	Mechanism of action	Health condition	Study	Phase	Status	Duration	*N*	Study design and drug doses	Primary outcomes	Results	Adverse Effects	Reference
Experimental mood stabilizers												
Ebselen	IMPase inhibitor	Mania or Hypomania	RCT	II	Completed	3 weeks	68	Add-on to TAU, In/outpatients, placebo-controlled, Ebselen:600 mg bd	YMRS	No statistical difference with placebo in ↓YMRS	Comparable to placebo	Sharpley et al. [34]
Valnoctamide	IMPase inhibitor	Mania	RCT	II	Completed	3 weeks	173	Monotherapy, three-armed (71=valnoctamide, 32=risperidone, 70=placebo) Valnoctamide:1500 mg/d	YMRS	No statistical difference with placebo in ↓YMRS	Comparable to placebo	Weiser et al. [35]
Eslicarbazepine	Voltage-dependent sodium channel inhibitor	Mania	RCT	II	Completed	3 weeks	160	Monotherapy, placebo-controlled, parallel group, dose-titration Eslicarbazepine up to 2400 mg/d	YMRS	No statistical difference with placebo in ↓YMRS	Slightly superior to placebo (dizziness, headache, nausea)	Grunze et al. [36]
Re-labelling antipsychotics												
Brexpiprazole	D2 par ago	Mania	RCT	III	Completed	3 weeks	322	Monotherapy, placebo-controlled	YMRS	No statistical difference with placebo in ↓YMRS	Akathisia	Vieta et al. [37]
	5HT1A par ago		RCT	III		3 weeks	332	Brexpiprazole: 2–4 mg/d				
	5HT2A antago		OL	III		26 weeks	381	Monotherapy, placebo-controlled, dose n/a				
Iloperidone	D2 antago 5HT2A antago	Mania	RCT	III	Ongoing (ends May 2023)	4 weeks	400 (est)	Monotherapy, placebo-controlled	YMRS	Pending	Pending	NCT04819776 [38]
Cariprazine	D2/D3 par ago	Maintenance BD	RCT	III	Ongoing (ends July 2023)	52 weeks	822	Cariprazine: 1.5–3 mg/d	Number of days to first relapse	Pending	Pending	NCT03573297 [39]
Experimental Antipsychotics												
ALKS3831 (OLZ + SAM)	OLZ: D2 antago 5HT2A antago SAM: μ-opioid antago	Acute Schizophrenia	RCT	III	Completed	4 weeks	352	Monotherapy, olanzapine and placebo-controlled, OLZ = 10–20 mg; SAM = 10 mg	PANSS-tot, CGI-S	OLZ + SAM > placebo in ↓ PANSS-tot and CGI-S	Weight gain, dry mouth, somnolence, anxiety, headache	Potkin, Kunovac, Silverman et al. [100]
		Chronic Schizophrenia	RCT	III	Completed	24 weeks	538	Outpatient, Monotherapy, olanzapine-controlled to assess safety profile	Body weight>10% at week 24	OLZ + SAM < OLZ in body weight gain	Weight gain, dry mouth, somnolence, increased appetite	Correll et al. [43]
								OLZ = 10–20 mg; SAM = 10 mg				
		BD-I, Schizophrenia	OL	III	Ongoing (ends Dec 2022)	Up to 48 months	500 (est)	Long-term safety and efficacy, dosing n/a	Incidence of treatment-emergent adverse events	Pending	Pending	NCT03201757 [41]
KarXT (xanomeline + trospium)	Xanomeline: Cholinergic M2 ago Trospium: peripheral M antagonist	Acute Schizophrenia	RCT	II	Completed	5 weeks	182	Monotherapy, placebo-controlled	PANSS-tot	KarXT>Placebo in ↓ PANSS-tot	constipation, nausea, dry mouth, dyspepsia, vomiting	Brannan et al. [44]
								Xanomelin up to 125 mg bd; Trospium up to 30 mg bd				
			OL	III	Ongoing (ends July 2023)	56 weeks	400 (est)	Open-label Study to Assess the Long-term Safety, Tolerability, and Efficacy, dosing of KarXT same as above	Incidence of treatment-emergent adverse events	Pending	Pending	NCT04820309 [45]
SEP-363856	TAAR1 ago 5HT1A ago	Acute Schizophrenia	RCT	II	Completed	4 weeks	245	Monotherapy, placebo-controlled SEP-363856: 50–75 mg/d	PANSS-tot	SEP-363856>Placebo in ↓ PANSS-tot	Somnolence and GI symptoms	Koblan et al. [46]
			RCT+OL	II/III	Ongoing (ends June 2025)	RCT: 6 OL: 12 weeks	480 (est)	Same as above	PANSS-tot	Pending	Pending	NCT04825860 [47]
RO6889450 (Ralmitaront)	TAAR1 par ago	Acute Schizophrenia or Schizoaffective Disorder	RCT	II	Ongoing (ends Apr 2023)	4 weeks	308 (est)	Monotherapy, placebo and risperidone-controlled Ralmitaront: 45–150 mg/d Risperidone: 4 mg/d	PANSS-tot	Pending	Pending	NCT04512066 [49]
Lumateperone	5HT2A antago, D2 par ago, glutamate (NMDA GluN2B) enhancer	Acute Schizophrenia	RCT	II	Completed	4 weeks	311	Monotherapy, placebo and risperidone-controlled; Lumateperone: 42-84 mg/d Risperidone: 4 mg	PANSS-tot	Only lumateperone 42 mg>Placebo in ↓ PANSS-tot	Headache, somnolence, dizziness	Greenwood et al. [101]
		Acute Schizophrenia	RCT	III	Completed	4 weeks	450	Monotherapy, placebo-controlled Lumateperone: 28–42 mg	PANSS-tot	Only lumateperone 42 mg>Placebo in ↓ PANSS-tot	Headache, somnolence, dizziness	Greenwood et al. [101]
		Bipolar Depression	RCT	III	Various Completed & Ongoing							D’souza et al. [51], NCT03249376 [102]
Brilaroxazine	Serotonin-dopamine modulator	Acute Schizophrenia and Schizoaffective Disorder	RCT	II	Completed	4 weeks	234	Monotherapy, placebo and aripiprazole-controlled Brilaroxazine: 15, 30, 50 mg/d Aripiprazole: 15 mg	PANSS-tot	Brilaroxazine>Placebo in ↓ PANSS-tot for 15 mg and 50 mg/d	Insomnia, agitation	Cantillon et al. [55]
F17464	D3 antago 5-HT1A par ago	Acute Schizophrenia	RCT	II	Completed	6 weeks	134	Monotherapy, placebo-controlled F17464: 20 mg bd	PANSS-tot	F17464 > Placebo in ↓ PANSS-tot	Insomnia, agitation, increased triglycerides	Bitter et al. [103]
AVN-211	5HT6 antago	Chronic Schizophrenia	RCT	II	Completed	4 weeks	42	Add-on to SOC, placebo-controlled AVN-211: 0.05–0.2/kg/d	PANSS, CGI-S, WAIS	AVN-211>Placebo in ↓ PANSS-positive score, CGI-S and ↑WAIS score	n/a	Capuzzi et al. [104]
Cannabidiol (CBD)	CB1 par ago 5HT1A par ago	Chronic Schizophrenia	RCT	II	Completed	6 weeks	88	Add-on to SOC, placebo-controlled CBD: 1000 mg/d	PANSS, CGI-I, CGI-S, GAF, BACS	CBD > Placebo in ↓ PANSS-positive score, CGI-I, CGI-S CBD failed to separate from placebo in GAF, BACS, PANSS-tot, negative and GP scores	Comparable to placebo	McGuire et al. [105]
		Mania	CS	n/a	Completed	5 weeks	2	Monotherapy, CBD: 600–1200 mg/d	YMRS	No evidence of efficacy	Well tolerated	Zuardi et al. [60]
Sodium Nitroprusside (SN)	Nitric Oxide donor	Acute Schizophrenia	RCT	n/a	Completed	4 weeks	20	Add-on to SOC, placebo-controlled SN: 0.5 μg/kg/min for 4 h	BPRS-18	SN > Placebo in ↓ BPRS-18-tot	Well tolerated	Hallak et al. [62]
		Acute Schizophrenia	RCT	n/a	Completed	4 weeks	42	Same as above	PANSS	No statistical significance	Well tolerated	Wang et al.2018 [64]
		Acute Schizophrenia	RCT	n/a	Completed	4 weeks	20	Same as above	BPRS-18 PANSS	No statistical significance	Well tolerated	Stone et al.2016 [63]
		Acute Schizophrenia	RCT	n/a	Completed	2 weeks	52	Same as above	PANSS	No statistical significance	Well tolerated	Brown et al. [65]
MK-8189	PDE10A inhibitor	Acute Schizophrenia	RCT	II	Completed	4 weeks	224	Monotherapy, placebo and risperidone-controlled, MK-8189 up to 12 mg/d Risperidone up to 6 mg	PANSS	No statistical difference with placebo in ↓PANSS-tot	n/a	NCT03055338 [66]
		Acute Schizophrenia	RCT	II	Ongoing (ends July 2022)	6 weeks	576	MK-8189 up to 24 mg/d Risperidone up to 6 mg	PANSS	Pending	Pending	NCT04624243 [67]
BI 409306	PDE9A inhibitor	Attenuated Psychosis Syndrome	RCT	II	Completed	52 weeks	50	Monotherapy, placebo-controlled, dose n/a	Time to remission	Pending	Pending	NCT03230097 [69]
Pimavanserin (PIM)	5-HT2A inverse ago	Acute Schizophrenia	RCT	III	Completed	6 weeks	423	Add-on to risperidone or haloperidol, placebo-controlled	PANSS	PIM + risperidone2mg equally efficacious as risperidone 6 mg in ↓PANSS-tot	Headache, Somnolence, Insomnia	Meltzer et al.2012 [70]
								PIM: 20 mg Risperidone: 2–6 mg Haloperidol: 2 mg				
		Treatment-resistant Schizophrenia	RCT	III	Completed	6 weeks	396	Add-on to SOC, placebo-controlled	PANSS	No statistical difference with placebo in ↓PANSS-tot	Well tolerated	NCT02970292 [71]
								PIM: 10, 20, 34 mg/ d				
		Negative Symptoms in Schizophrenia	RCT	III	Ongoing (ends Mar 2023)	26 weeks	426	Add-on to SOC, placebo-controlled	NSA-16	Pending	Pending	NCT04531982 [106]
								PIM: 34 mg/d				
Evenamide	Glutamate modulator and voltage-gated sodium channel blocker	Acute Schizophrenia	RCT	II	Completed	4 weeks	89	Add-on to risperidone or aripiprazole, placebo-controlled	PANSS	Evenamide>placebo In ↓PANSS-tot	Well tolerated	Anand et al. [74]
								Evenamide: 15, 25 mg/d Risperidone: 4 mg/d Aripiprazole: 20 mg/d				
		Acute Schizophrenia	RCT	II	Completed	4 weeks	138	Add-on to SOC, placebo-controlled	PANSS	Pending	Pending	NCT04461119 [75]
								Evenamide: 7,5 - 15 mg bd				
Experimental non-mood-stabilizer/ non-antipsychotic agents												
Endoxifen	PKC inhibitor	Mania or mixed state	RCT	III	Completed	3 weeks	84	Monotherapy, divalproex-controlled Endoxifen: 4–8 mg/d	YMRS	Endoxifen faster than divalproex in ↓ YMRS	Well tolerated as compared to divalproex	Ahmad et al. [78]
		Mania	RCT	III	Completed	3 weeks	228	Monotherapy, divalproex-controlled Endoxifen: 8 mg/d	YMRS	Endoxifen faster than divalproex in ↓ YMRS	Headache, insomnia, vomiting	Ahmad et al. [79]
		Mania	RCT	III	Completed (ended May 2021)	3 weeks	124	Monotherapy, placebo-controlled Endoxifen: 8 mg/d	YMRS	Pending	Pending	NCT04315792 [107]
Tamoxifen (TMX)	PKC inhibitor	Mania or Hypomania	PS	n/a	Completed	4 weeks	13	Monotherapy, placebo-controlled TMX: 40 mg/d	CARS-M	TMX > placebo in ↓ CARS-M	n/a	Kulkarni et al. [108]
		Mania or Mixed State	RCT	III	Completed	3 weeks	16	Monotherapy, placebo-controlled TMX: 20–140 mg/d	YMRS	TMX > placebo in ↓ YMRS	Loss of appetite	Zarate et al. [109]
		Mania or Mixed State	RCT	III		3 weeks	50	Monotherapy, placebo-controlled TMX: 80 mg/d	YMRS	TMX > placebo in ↓ YMRS	Comparable to placebo	Yildiz et al. [110]
		Mania	RCT	III	Completed	6 weeks	40	Add-on to lithium, placebo-controlled TMX: 80 mg/d	YMRS, PANSS	TMX > placebo in ↓ YMRS and PANSS	Fatigue	Amrollahi et al. [111]
		Mania	RCT	III	Completed Completed	4 weeks	51	Add-on to SOC, placebo-controlled	CARS-M	No statistical difference	Comparable to placebo	Kulkarni et al. [112]
Celecoxib	COX-2 inhibitor	Mania without psychotic symptoms	RCT	III	Completed	6 weeks	46	Add-on to divalproex, placebo-controlled	YMRS	Celecoxib> placebo in ↓ YMRS	Comparable to placebo	Arabzadeh et al. [81]
								Celecoxib: 400 mg/d				
		Mania without psychotic symptoms	RCT	III	Completed	8 weeks	40	Add-on to lithium+risperidone, placebo-controlled	YMRS	Celecoxib> placebo in ↓ YMRS	Comparable to placebo	Mousavi et al. [82]
								Celecoxib: 200 mg/d				
N-acetylcisteine (NAC)	Multi-target molecule with anti-oxidant properties	Mania or Hypomania	RCT (post-hoc analysis)	III	Completed	24 weeks	15	Add-on to TAU, placebo-controlled NAC: 1000 mg bd	YMRS	NAC > placebo in ↓ YMRS	Comparable to placebo	Magalhães et al. [83]
Omega-3 polynsaturated fatty acids	Arachidonic acid competitor	Mania	RCT	III	Completed	4 weeks	14	Add-on to divalproex, placebo-controlled Omega3: EPA 440 mg +DHA 240	YMRS, PANSS	No statistical difference	Comparable to placebo	Chiu et al. [84]
Probiotics (*Lactobacillus rhamnosus* strain GG and *Bifidobacterium animalis* subsp. lactis strain Bb12)	Regulation of gut-microbiota	Remitted Mania	RCT	III	Completed	24 weeks	66	Add-on to TAU, placebo-controlled Probiotics: 10^9^ CFU	Rehospitalization index, days rehospitalized	Probiotics>placebo in rehospitalization index and days rehospitalized	Comparable to placebo	Dickerson et al. [85]
		Remitted Mania	RCT	III	Ongoing (end March 2022)	24 weeks	66	Same as above	Same as above	Pending	Pending	NCT03383874 [86]
Methylphenidate	Noradrenaline and Dopamine reuptake-inhibitor	Mania	RCT	III	Completed	2.5 days	42	Monotherapy, placebo-controlled	YMRS	No statistical difference	Well tolerated	Hegerl et al. [87]
								Methylphenidate: 20–40 mg/d				
Memantine	Glutamate NMDA channel blocker	Mania	OL	n/a	Completed	4 weeks	35	Monotherapy Memantine: 20–40 mg/d	YMRS	Signs of efficacy in ↓ YMRS	Constipation, nausea, headache	Keck et al. [113]
		Mania (patients > 60 years old)	RCT	III	Completed	8 weeks	70	Add-on to divalproex, placebo-controlled Memantine: 5–20 mg/d	YMRS	Memantine> placebo in ↓ YMRS		Omranifard et al. [90]

*IMP* Inositol-Mono-Phosphatase; *RCT* Randomized Clinical Trial, *YMRS* Young Mania Rating Scale, *TAU* Treatment As Usual, *OL* Open label, *est* estimated, *n/a* not available, *OLZ* Olanzapine, *SAM* Samidorphan, *PANSS* Positive and Negative Syndrome Scale, *tot* total score, *pos* positive symptoms score, *neg* negative symptoms score, *CGI-S* Clinical Global Impression-Severity scale, *TAAR* Trace-amino-associated receptor, *GI* Gastro-intestinal, *SOC* Standard of care, *CGI-I* Global Clinical Impression - Improvement scale, *GAF* Global Assessment of Functioning scale, *BACS* Brief Assessment of Cognition scale, *GP* General Psychopathology, *CS* Case-series, *BPRS-18* Brief Psychiatric Rating Scale 18 item version, * especially negative symptoms, *SANS* Scale for Assessment of Negative Symptoms, *QLS* Quality of life scale, *MCCB* MATRICS Consensus Cognitive Battery, *DAAO* d-amino acid oxidase, *PKC* Protein-Kinase C, *COX* Cyclo-Oxygenase, *CARS-M* Clinical-Administered Rating Scale for Mania, *PS* Pilot study, *EPA* Eicosapentanoic acid, *DHA* docosahexaenoic acid, *CFU* Colony-Forming Unit.

As mentioned above, regarding emerging and experimental approaches to mania three main issues should be considered: (a) experimental research is poorly dedicated specifically to the psychopharmacology of manic episodes; (b) the consequent—whether motivated—need for looking at the field of experimental psychopharmacology in Schizophrenia in order to bridge the gap in the experimental research of psychopharmacology of manic episodes and (c) in both BD and Schizophrenia, current research is focused more on overcoming safety and tolerability side effects of existing drugs rather than potentiating their efficacy profiles.

### Experimental mood stabilizers

Three experimental MSs are listed in Table 2.

Ebselen: this compound has been chosen as an experimental MS for its strong activity as inositol monophosphatase (IMPase) inhibitor, one of the putative pharmacological targets of lithium [33]. In a recent phase IIa, double-blind, placebo-controlled, add-on randomized clinical trial (RCT) with in/outpatients suffering from mania or hypomania, Ebselen was not statistically superior to placebo in reducing manic symptoms after 3 weeks of treatment, even though descriptive data indicated a tendency towards superiority [34]. The two groups did not differ significatively in terms of adverse effects. The authors concluded that Ebselen deserves further research in which concomitant psychotropic treatment is better controlled [34].

Valnoctamide: this divalproex derivative with demonstrated IMPase inhibition activity but no demonstrated activity in inhibiting voltage-gated sodium channels, was tested as monotherapy for acute mania and compared to placebo and risperidone in one RCT [35]. Valnoctamide was supposed to have a better reproductive tolerability profile than Divalproex since it showed decreased risk for congenital abnormalities in animals. The results of the RCT showed lack of efficacy for this agent. Investigators hypothesized that the lack of efficacy could be due to the above-mentioned pharmacodynamic differences between Valnoctamide and Divalproex [35].

Eslicarbazepine: is an oxcarbazepine-like anticonvulsant agent, approved in 2009 for treatment of partial onset seizures. In a multicenter, double-blind RCT, Eslicarbazepine was tested in patients with acute mania in search of an effective alternative to MSs. As in the two precedent cases, results showed lack of efficacy in primary outcomes [36].

### Re-labelling antipsychotics

Three agents are listed in Table 2.

Brexpiprazole is a dopamine D2 receptor partial agonist and serotonergic 5HT1A partial agonist/5HT2A antagonist, is already indicated as monotherapy for Schizophrenia and as adjunctive treatment to antidepressants for Major Depressive Disorder. In pre-approval trials, Brexpiprazole showed signs of a better safety/tolerability profile than other drugs of its same class (namely aripiprazole, cariprazine) so it was recently tested as treatment for acute mania in two double-blind placebo-controlled and one open-label long-term RCT [37]. The results of the trials indicated a lack of statistically significant difference in terms of efficacy between brexpiprazole and placebo. Possible explanations for this failure may include slow titration/ modest dose schedules and samples’ heterogeneity, especially with respect to psychopathological features (above all insight) and standards of care (hospitalization vs outpatient treatment) [37].

Iloperidone is a dopamine/serotonin receptor antagonist (D2, 5HT2A) approved in 2009 by the Food and Drug Administration (FDA) for use in Schizophrenia. However, iloperidone did not received the marketing authorization by the European Medicines Agency (EMA) in 2018, mainly because of worries about QTc prolongation risk [https://www.ema.europa.eu/en/documents/assessment-report/fanaptum-epar-refusal-public-assessment-report_en.pdf]. Nonetheless, Iloperidone is considered to show a good safety/tolerability profile in comparison with other antipsychotics, especially on the metabolic and neurological sides [38]. It is currently being tested in patients with acute mania in a multicenter double-blind placebo-controlled RCT, expected to be completed in May 2023 [39].

Cariprazine is a dopamine D3 > D2 partial agonist, already approved by the FDA for the treatment of bipolar mania and bipolar depression and by the EMA for the treatment of schizophrenia. It is currently under study in a placebo-controlled RCT to evaluate its long-term safety/tolerability profile and its efficacy in preventing relapses of both mania and depression in BD [40]. In case of yielding positive results, cariprazine could improve its evidence base as a treatment for all phases of BD.

### Experimental antipsychotics

ALKS3831 is a combination of olanzapine plus samidorphan, a new chemical entity that acts as a μ-opioid modulator. ALKS3831 is currently under development in phase III trials to test its efficacy and safety in patients with Schizophrenia and BD-I [41]. The adjunct of samidorphan was primarily intended to mitigate weight gain, a well-known side effect of olanzapine, while useful secondary outcomes of this combination might include reduced craving for alcohol and mood regulation via the opiod system [42]. In a recent phase III trial in patients with schizophrenia, a combination of different doses of olanzapine/samidorphan was double-blindly compared to olanzapine alone. While retaining a similar antipsychotic efficacy to olanzapine, the combined treatment proved to be effective in reducing olanzapine-induced weight-gain [43].

KarXT is a combination of xanomeline, an experimental muscarinic M2 receptor cholinergic agonist, and trospium chloride, an already marketed agent acting peripherally as a muscarinic cholinergic antagonist. Novelty features about KarXT mainly consist in targeting the cholinergic system without any anti-dopaminergic activity. This minimizes the risk for dopamine-system-related side-effects, common to many FGAs and SGAs. Regarding the putative use of central cholinergic agonists in BD, one should be aware of previous literature suggesting the presence of low levels of central acetylcholine in mania and high levels in depression [12]. While central cholinergic agonists are likely to reduce manic symptoms, the risk for counterpolar switch or the emergence of depressive symptoms should be taken in consideration and accurately monitored. KarXT completed a phase II trial in patients with Schizophrenia, showing statistically significant efficacy in reducing psychotic symptoms. However, adverse events related to cholinergic and anti-cholinergic activity (constipation, nausea, dry mouth, dyspepsia, and vomiting) were significatively more present in the active drug group compared with the placebo group [44]. Investigators conclude that larger and longer studies are needed to better specify the efficacy and safety profiles of KarXT. One open-label study to assess the long-term safety, tolerability and efficacy of KarXT in de novo subjects with Schizophrenia is ongoing [45] and expected to complete in July 2023.

Another non-dopaminergic agent under study in psychotic disorders is SEP-363856, a trace amine–associated receptor 1 (TAAR1) agonist and serotonin 5HT1A receptor agonist. It completed the phase II placebo-controlled RCT in 120 patients with an acute exacerbation of schizophrenia and proved to be effective in lowering psychotic symptoms and a good short-term safety/tolerability profile (more common adverse effects included somnolence and gastrointestinal symptoms but no differences in extrapyramidal and glucose-lipid metabolism were found between groups) [46]. SEP-363856 is currently under study in one placebo-controlled RCT to continue evaluating its efficacy and safety in patients with schizophrenia, followed by one open-label extension phase expected to be completed in June 2025 [47].

RO6889450 (or Ralmitaront) is another agent acting on TAAR1, with a partial agonist profile [48]. To our knowledge, phase II study results for Ralmitaront have not been published. One phase II trial of the efficacy and the safety of Ralmitaront vs placebo and risperidone in patients with an acute exacerbation of schizophrenia or schizoaffective disorder is currently ongoing, expected to complete in April 2023 [49].

Currently under-study antipsychotic agents with dopaminergic activity include Brilaroxazine (RP5063), F17464 and FKF02SC, while Lumateperone (ITI-007) was finally approved by the FDA in December 2019 as a novel agent for the treatment of schizophrenia.

Lumateperone simultaneously modulates serotonin, dopamine and glutamate (5HT2A antagonist, D2 partial agonist, NMDA-GluN2B enhancer). In pre-approval clinical trials, it demonstrated efficacy in lowering psychotic symptoms during acute schizophrenia and a good short-term safety/tolerability profile with only somnolence/sedation indexes significantly higher than placebo [50]. Lumateperone’s pharmacodynamic profile suggested its hypothetical efficacy in bipolar depression and the results coming from the first trials tend to confirm the hypothesis [51–53]. No clinical trials of lumateperone in bipolar mania have been performed to our knowledge.

Brilaroxazine is a serotonin-dopamine modulator, structurally similar to aripiprazole, brexpiprazole, and cariprazine [54]. In a phase II double-blind, placebo and aripiprazole-controlled clinical trial, brilaroxazine demonstrated efficacy in reducing psychotic symptoms and signs of a good safety/tolerability profile: no significant changes in body weight, electrocardiogram, or incidence of orthostatic hypotension, decrease in blood glucose, lipid profiles, and prolactin levels. Aripiprazole was included only to demonstrate assay sensitivity and was not powered to show efficacy [55]. No ongoing clinical trials with brilaroxazine were found in the FDA and EMA’s registers.

F17464, a dopamine D3 antagonist/serotonin 5HT1A partial agonist, is under study as monotherapy for schizophrenia. It completed one phase II double-blind, placebo-controlled RCT where it proved to be effective in lowering Positive and Negative Syndrome Scale (PANSS) total, positive and general psychopathology subscores but not PANSS negative subscore, with a favorable safety profile [48, 56]. To the best of our knowledge, no clinical trials involving F17464 are ongoing.

AVN-211 is an experimental serotonin 5HT6R antagonist. In a pilot phase II, add-on, placebo-controlled RCT in schizophrenia patients, AVN-211 demonstrated efficacy in lowering PANSS positive and CGI-S scales [57]. It is currently under development according to producer Avineuro, but no ongoing trials were found in FDA or EMA’s registers.

Cannabidiol (CBD), an agent acting on a variety of neuronal receptors including CB1 (partial agonism) and serotonin 5HT1A (partial agonism), is currently under study for psychotic disorders in several clinical trials [58, 59]. Due to its receptor binding profile, it is supposed to show antipsychotic and procognitive properties [48]. In an exploratory, add-on, placebo-controlled clinical trial in patients with schizophrenia, CBD demonstrated efficacy in improving the Clinical Global Impression (CGI) score and in lowering PANSS positive subscore, but did not significatively differed from placebo in the Global Assessment of Functioning (GAF) and total, negative and general psychopathology PANSS scores [48]. Regarding the use of CBD in acute mania, just one small case series [60] exists, in which CBD showed no efficacy [61].

Sodium Nitroprusside acts as a Nitric Oxide (NO) donor, a gas that could be involved in the pathophysiology of schizophrenia by mediating the release of neurotransmitters [48]. Although an original, exploratory, placebo-controlled study in acutely ill schizophrenic patients showed significant superiority of sodium nitroprusside over placebo in reducing psychotic symptoms [62]. However, three subsequent studies did not confirm these prior results [63–65].

Two phosphodiesterase (PDE) inhibitors are listed in Table 2.

MK-8189 is a PDE10A inhibitor, expected to target both dopaminergic and glutamatergic activity in the striatum via cAMP, cGMP and phosphorylation-dependent mechanisms [48]. However, in one placebo and risperidone-controlled RCT with acutely ill schizophrenic patients, MK-8189 did not separate from placebo on efficacy outcome measures, while risperidone did [66]. Another similarly designed clinical trial with MK-8189 is actually ongoing and expected to complete in July 2022 [67].

BI 409306 is a PDE9A inhibitor whose putative mechanism involves glutamatergic regulation via cGMP-dependent mechanisms. This drug was tested in a phase II placebo-controlled clinical trial as add-on for cognitive symptoms in schizophrenia in which it did not separate from placebo in primary outcomes [68]. Now it is under study as an early intervention monotherapy for attenuated psychosis syndrome [69]. The study actually completed in April 2021, but results are still unpublished.

Pimavanserin is a serotonin 5HT2A inverse agonist, approved for the treatment of delusions and hallucinations in patients with Parkinson’s Disease. In one prior study pimavanserin was tested as augmentation therapy to risperidone for schizophrenia [70] and more recently as an add-on to background AP for treatment resistant schizophrenia [71]. While in the first study pimavanserin proved effective in augmenting risperidone’s antipsychotic potential with a good safety/tolerability profile, in the second one it did not reach statistical significance over placebo in lowering PANSS scores. Pimavanserin is currently under study as an add-on therapy for negative symptoms in schizophrenia [72]. The RCT is expected to be completed in March 2023.

Evenamide is an experimental agent with completed and ongoing phase II RCT in patients with Schizophrenia. Its mechanism of action includes voltage-gated sodium channel blockade and glutamate modulation [73]. In a phase II placebo-controlled RCT, Evenamide showed evidence of antipsychotic efficacy as an add-on to risperidone or aripiprazole, with no signs of dopaminergic or serotoninergic activity [74]. In March 2021, Evenamide completed another phase II, placebo-controlled RCT in patients with schizophrenia who were symptomatic on their SGA medication, but results of the study are still unknown [75]. Regarding a hypothetical treatment of bipolar mania with Evenamide, some promising features could be found in its mechanism of action (sodium channel blockade shared with MS like Divalproex, while adding glutamate regulation) and its demonstrated efficacy in lowering both PANSS total score and PANSS positive subscore. However, larger, longer and specifically designed RCT are needed to validate this hypothesis.

### Non-Mood stabilizer/non-Antipsychotic emerging treatments

Multiple factors interact in the complex equation of the pathophysiology of a ME. As a consequence, beyond neurotransmission and signal transduction, also neuro-hormonal pathways, oxidative stress and changes in the immune system are considered as putative pharmacological targets for the treatment of a ME.

#### Tamoxifen and endoxifen

Tamoxifen is an estrogen antagonist whose mood-regulating properties are supposed to be mediated via phosphokinase C (PKC) inhibition [76]. A recent metanalysis [77] found 5 RCT of tamoxifen in acute manic patients, 3 ass add-on and 2 as monotherapy. Results of the studies homogenously indicate evidence of efficacy for tamoxifen over placebo in reducing manic symptoms. Endoxifen, an active metabolite of tamoxifen with a four-fold PKC inhibitory activity compared with tamoxifen, was recently tested in a phase III double-blind, divalproex-controlled RCT with acutely manic patients. The trial demonstrated efficacy of endoxifen in reducing manic symptoms and faster action as compared to divalproex with a good short-term adverse events profile [78, 79].

However, concerns about long-term side-effects limits the approval of these agents for clinical practice. Larger and longer RCT are needed to demonstrate long-term safety/tolerability reliability of PKC inhibitors for BD patients.

#### Antinflammatory and antioxidant drugs

Inflammation, oxidative stress, and mitochondrial dysfunction have been identified as key factors in the pathophysiology and neuroprogression of BD [11]. As a consequence, antinflammatory and antioxidant agents have been tested for efficacy in acute bipolar mania in recent years [80].

Regarding the use of anti-inflammatory drugs, Celecoxib was administered in two placebo-controlled RCT as add-on to Divalproex [81] or Lithium plus Risperidone [82] in patients with acute mania without psychotic symptoms. In both trials, celecoxib demonstrated significant efficacy in reducing manic symptoms compared to placebo. No ongoing clinical trials for treatment of bipolar mania with celecoxib or other antiflammatory drugs were found in FDA or EMA registers.

N-acetylcysteine (NAC), a multi-target molecule with antioxidant properties, presented signs of efficacy in one post-hoc analysis with a subset of manic patients from a placebo-controlled RCT [83] with a small sample size (*n* = 15). Larger and specifically designed RCT are needed to validate hypothesis of efficacy.

Omega-3s are antinflammatory molecules competing with arachidonic acid, thereby decreasing the production of pro-inflammatory eicosanoids. One small RCT (*n* = 15) evaluated the effects of add-on omega-3s in acute mania, finding no significant differences in manic symptoms compared to placebo [84].

#### Probiotics

Regulation of gut microbiota has been targeted in BD investigation with the aim of testing pro-cogntive and mood-regulating effects of gut-brain signaling [12]. One placebo-controlled RCT was performed in patients discharged following hospitalization for acute mania [85]. The results showed efficacy for probiotics (*Lactobacillus rhamnosus* strain GG and *Bifidobacterium animalis* subsp. lactis strain Bb12) over placebo in delaying time to rehospitalization and reducing length of stay in psychiatric ward. Another probiotic intervention to prevent relapse following hospitalization for bipolar mania is currently ongoing and expected to complete in September 2021 [86].

#### Methylphenidate

Methylphenidate, a noradrenaline-dopamine reuptake inhibitor already approved in the US and EU for use in Attention-Deficit Hyperactivity Disorder and Narcolepsy, was recently tested in acute manic patients under the hypothesis that it could influence wakefulness with a mood-regulating outcome. The MEMAP study, a large multi-center placebo-controlled RCT concluded in September 2016, demonstrated a lack of efficacy for methylphenidate over placebo in acute mania [87].

#### Memantine

Lastly, Memantine is a glutamate NMDA receptor blocker, actually approved for use in moderate to severe Alzheimer’s disease but with a good amount of literature suggesting its use in other psychiatric disorders [88] and particularly bipolar disorder [89]. In a recent placebo-controlled RCT, conducted in Iran on a sample of 70 elderly patients with acute mania, memantine showed some efficacy as add-on to valproate in reducing manic symptoms and improving cognition over placebo [90].

## Discussion and conclusion

The treatment of mania still presents very important challenges, especially when the long-term is considered. Although the pharmacological armamentarium to treat this condition currently list a good number of drugs, the gap between evidence-based treatment and clinical practice is still considerable, and the majority of patients need combined pharmacological therapies and psychosocial interventions to achieve reasonable results in terms of clinical and functional outcomes.

Treatment of acute mania should be personalized in consideration of several clinical criteria, which include manic episode presentation features (psychotic, mixed, and anxious features), course specifiers (PP and rapid cycling), and patient factors (disease severity, comorbidities, treatment adherence, present and past treatment, and the stage of disease).

Another aspect to take into consideration is that for ethical reasons most of the evidence comes from studies where participants agree to participate and sign an informed consent to be enrolled in the study. As a consequence, they could present milder forms of mania in comparison with the patients commonly attended in clinical practice. For this reason, doses recommended in guidelines tend to be lower than those used in clinical practice and guidelines are also more supportive of monotherapy, while polytherapy is the rule in the real world.

Regarding the emerging approaches, the paucity of recent or ongoing trials specifically targeting the treatment of mania should be remarked. Experimental MSs we discussed in section Existing approaches to the treatment of mania did not reach statistical significance of efficacy in acute mania and no ongoing trial is being conducted to replicate or modify those initial negative results. In addition, few expectations are linked to the effort of re-labelling existing APs for use in bipolar mania. Although probably influenced by some flaws in the design of the trial, Brexpiprazole recently failed to demonstrate efficacy in acute mania while for Iloperidone—which is marketed in the US but not in the European countries—results are waited in May 2023. Among novel and experimental APs, the most promising agents under study for schizophrenia (e.g. KarXT, SEP-363856, RO6889450, ALKS3831, Evenamide) could be hypothetically considered to show benefits also for bipolar mania in consideration of their pharmacodynamic characteristic, antipsychotic efficacy and safety/tolerability profile. As we have already remarked, their main benefits in the field of psychopharmacotherapy are expected to come in terms of a better safety/tolerability profile than existing agents. As for safety/tolerability, regarding a putative use of these agents in the treatment of a ME, it may be again remarked that central cholinergic agonism exerted by KarXT should be accurately monitored for risk of counterpolar depressive switch. SEP-363856, RO6889450 might not present this risk. Lastly, ALKS3831 could ameliorate the already demonstrated efficacy of olanzapine in patients with BD, by improving its long-term metabolic safety/tolerability profile. Among non MS/non AP agents, PKC inhibitors (especially endoxifen) represent the class of drugs which proved to be capable of directly competing with approved agents in the treatment of mania, because demonstrated efficacy and a good short term safety/tolerability profile. However, concerns about their endocrinological activity make longer and larger RCT still necessary before considering their use in clinical practice. Other types of agents, like antinflammatories/antioxidants or probiotics, demonstrated some level of efficacy as add-on to standard agents for reducing manic symptoms (celecoxib) or prolongate time to re-hospitalization after a manic episode (probiotics).

In addition, *re-emerging* approaches in the pharmacotherapy of BD—a topic which has gained attention in BD scientific literature in the last year [12, 91, 92]—should also be highlighted. The last few decades have seen a dramatic increase in available treatments for mania [93] and this has been associated with shifts in prescribing patterns with a reduction in the use of mood stabilizers and an increase in the use of antipsychotics [94]. However, despite the increase in available treatment options, there has also been a documented increase in dysfunction and disability in patients with bipolar illness [95]. This led some influencing authors in the field of BD research to advocate for the re-emergence of MSs’ prescription and to “Make Lithium Great Again!” [14]. While the validity of this argument is supported by several authors [91, 92], it is also important to intensify the efforts in the fields of experimental BD psychopharmacology and neurobiology in order to identify new treatment targets and pharmacological agents with better risk/benefits profiles.

Lastly, we would like to underline the importance of integrating pharmacological and non-pharmacological approaches in different stages of mania. A manic episode has to be seen as a severe episode of acute illness to be promptly treated, once its distinctive features are recognized, with the best personalized pharmacological approach, while keeping an eye on the long-term maintenance perspective. Once acute mania has remitted, prevention strategies such as behavioral and psychoeducation approaches should be implemented to lower the relapse risk [96]. These strategies include supporting continued treatment adherence, minimizing residual symptoms, early identifying signs of relapse, and promoting functional recovery [97–99].

### Limitations

The main limitations of this work are connected with its strengths. They include the non-systematic nature of the review. However, the aim of this piece was to provide a critical overview and some practical indications about current and emerging approaches to mania, informed by our clinical experience. In this respect, our group has prolonged experience over three decades of treating large numbers of manic patients and we follow a large cohort of bipolar patients, which has been the source of significative, valuable knowledge. Finally, conflicts of interest of the authors are listed in the specific section.
